# Antioxidant Enzyme Activities and Secondary Metabolite Profiling of Oil Palm Seedlings Treated with Combination of NPK Fertilizers Infected with* Ganoderma boninense*

**DOI:** 10.1155/2018/1494157

**Published:** 2018-03-12

**Authors:** Mahbod Sahebi, Mohamed M. Hanafi, Hasmah Mohidin, M. Y. Rafii, Parisa Azizi, Abu Seman Idris, A. Fariz, Rambod Abiri, Sima Taheri, Mehdi Moradpoor

**Affiliations:** ^1^Laboratory of Climate-Smart Food Crop Production, Institute of Tropical Agriculture and Food Security, Universiti Putra Malaysia, 43400 Serdang, Selangor, Malaysia; ^2^Laboratory of Plantation Science and Technology, Institute of Plantation Studies, Universiti Putra Malaysia, 43400 Serdang, Selangor, Malaysia; ^3^Department of Land Management, Faculty of Agriculture, Universiti Putra Malaysia, 43400 Serdang, Selangor, Malaysia; ^4^Faculty of Agrotechnology and Plantation, Universiti Teknologi MARA, Sarawak Branch, 94300 Kota Samarahan, Malaysia; ^5^Natural Product Research Development Centre, Universiti Teknologi MARA, Sarawak Branch, 94300 Kota Samarahan, Malaysia; ^6^Faculty of Agrotechnology and Plantation, Universiti Teknologi MARA, 40450 Shah Alam, Selangor, Malaysia; ^7^Biological Research Division, GanoDROP Unit, Malaysia Palm Oil Board, No. 6, Persiaran Institusi, Bandar Baru Bangi, 43000 Kajang, Selangor, Malaysia; ^8^Department of Biochemistry, Faculty of Biotechnology and Biomolecular Sciences, Universiti Putra Malaysia, 43400 Serdang, Selangor, Malaysia; ^9^Department of Crop Science, Faculty of Agriculture, Universiti Putra Malaysia, 43400 Serdang, Selangor, Malaysia

## Abstract

Oil palm (*Elaeis guineensis* Jacq) is one of the major sources of edible oil. Reducing the effect of* Ganoderma, *main cause of basal stem rot (BSR) on oil palm, is the main propose of this study. Understanding the oil palm defense mechanism against* Ganoderma* infection through monitoring changes in the secondary metabolite compounds levels before/after infection by* Ganoderma* under different fertilizing treatment is required. Oil palm requires macro- and microelements for growth and yield. Manipulating the nutrient for oil palm is a method to control the disease. The 3-4-month-old oil palm seedlings were given different macronutrient treatments to evaluate induction of defense related enzymes and production of secondary metabolite compounds in response to* G. boninense *inoculation. The observed trend of changes in the infected and uninfected seedlings was a slightly higher activity for *β*-1,3-glucanases, chitinase, peroxidase, and phenylalanine ammonia-lyase during the process of pathogenesis. It was found that PR proteins gave positive response to the interaction between oil palm seedlings and* Ganoderma* infection. Although the responses were activated systematically, they were short-lasting as the changes in enzymes activities appeared before the occurrence of visible symptoms. Effect of different nutrients doses was obviously observed among the results of the secondary metabolite compounds. Many identified/unidentified metabolite compounds were presented, of which some were involved in plant cell defense mechanism against pathogens, mostly belonging to alkaloids with bitter-tasting nitrogenous-compounds, and some had the potential to be used as new markers to detect basal stem rot at the initial step of disease.

## 1. Introduction

Oil palm (*Elaeis guineensis* Jacq.) is one of the world's sources of edible oil produced largely in Indonesia and Malaysia. Beside the palm oil and kernel oil, various products such as paper, pulp, particle boards, fertilizer, energy, and biofuels are derived from fiber, empty fruit bunches, and trunk of oil palm [1, 2]. Annually, the increasing demands for the oil palm products lead to expanding the cultured area of this tree in the major producing countries [3, 4].

Basal stem rot (BSR) disease caused by* Ganoderma boninense *is a serious threat for oil palm [5]. Reportedly, between 30 and 70% of oil palm production is usually lost because of BSR during repeating planting cycles [6]. The* Ganoderma *spp. attack the roots of palm, then spread slowly to the stem's bole, and cause dry rot that stops nutrient uptake and transport [7]. This infection leads to decrease in the ability of fruits producing and in the sever levels causes collapse of the trunk. Fruiting bodies of* Ganoderma *on the oil palm are not detectable till more than half of the internal tissues would be rotted [5]. Currently, research on biochemical and molecular aspects of oil palm at the early stage of the BSR disease is ongoing to understand interactions between palm and* Ganoderma*. In this regard, any changes in defense-responsive genes, transcription factors, proteins, and metabolite compounds should be monitored between healthy and* Ganoderma-*inoculated oil palm seedlings.

Plants have to develop their chemical-defense metabolites to stand unfavorable conditions and survive. These chemical-defense metabolites can be either resistance against pathogen or tolerance towards abiotic stress [8]. Therefore, induction of defense metabolite compounds in oil palm leaves may involve in tolerance of tree and acts as defense mechanism against* Ganoderma *[9]. Reportedly, the amount of secondary metabolite compounds in plants is affected by Phosphate (P) and Potassium (K) fertilizers [10–12]. It was proposed that production of cellulose, starch, and non-nitrogen containing secondary metabolite compounds and some of important bioactive compounds would be correlated and increased in well-fertilized crops [13]. Results of another study showed that application of combined biofertilizers, NPK fertilizer, and vermicompost obviously effected biochemical and morphophysiological responses of mustard plant. All these changes lead to synthesis and accretion of some secondary metabolite compounds; also some pigment contents, such as proline, sugar, and chlorophyll in plant leaves, resulted in regulating plant osmosis and growth improvement [14].

Effects of N, P, and K fertilizing on resistance of oil palm against* Ganoderma* via induction of fungi associated proteins need to be more considered. Comparison of metabolite compounds between NPK treated and untreated palms leaf extract is one of the methods to reveal the biochemical pathways induced by BSR disease. In the current research LC-MS as a metabolite profiling technique was applied to identify different metabolite compounds from leaves of two groups of NPK treated and untreated oil palm seedlings as another objective of this study. Profiling of these metabolite compounds will provide strong possibility to be used as biomarker discovery of BSR disease as well as understanding enhanced resistance of oil palm against* Ganoderma* [15].

## 2. Material and Methods

### 2.1. Site and Plant Material

The 3-4-month-old oil palm seedlings were obtained from the Federal Land Development Authority (FELDA) Agriculture Services Sdn Bhd, Sungai Tekam, Jerantut, Pahang, Malaysia. Seedlings were given treatments as presented in Tables 1 and 2 to evaluate induction of defense related enzymes as a response to macronutrients application on* G. boninense *inoculated and healthy oil palm seedlings. The experiment was conducted at the experimental farm (2°59′20.56^″^N, 101°42′44.42^″^E) under nursery shade house conditions. The experiment was designed as a split-plot with* Ganoderma* infected palms and uninfected palms as main plots and ten fertilizers treatment rates as subplots. Each main plot consisted of 500 healthy uninfected palms (−G) as control and 500* Ganoderma* (+G) infected palms. The treatment comprised low, optimum, and high level for N, P, K nutrition with respective element in mixed fertilizers. The experiment was conducted using a randomized complete block design (RCBD), with five replications of ten seedlings per treatment. The nutrient levels of fertilizer were represented by N1, P1, and K1 for “low”; N2, P2, and K2 for “optimum”; and N3, P3, and K3 for “high” level. The recommended optimum level (referred to as N2, P2, and K2) applied to the oil palm seedlings was formulated based on the best vegetative growth performance obtained from the previous experiment. Fertilizer treatment was applied once every four weeks and the other elements, such as magnesium and micronutrients serve as basic fertilizer (BF). The composition of different fertilizer treatments used is presented in Table 1.

The sources of macronutrients were straight fertilizer consisting of urea [CO(NH_2_)_2_] for N (46% N), triple superphosphate [Ca(H_2_PO_4_)_2_ H_2_O] for P (46% P_2_O_5_), and muriate of potash for K (60% K_2_O). The basic fertilizers (BF) were referred to as the other elements and they include MgO (source from kieserite) and trace elements as recommended in commercial production pocket guide in nursery stage [16]. The control, T10, was the granular compound fertilizer (CF), commonly used commercial fertilizer, where N stands for % N (12%), P for % P_2_O_5_ (12%), K for % K_2_O (17%), and Mg for % MgO (2%).

### 2.2. Experimental Design and Treatment

Based on the pathological performances and occurrence of diseases incidence responses, treatments T2, T5, T6, T8, and T10 were selected to be further evaluated of their enzymes activities. Treatments T2, T5, T6, T8, and T10 were arranged in a randomized complete block design with five replications of 20 seedlings per experimental unit with* G. boninense *infected and healthy palms for the detection test. Biochemical investigations were performed using three seedlings per treatment at weekly interval. Plants were destructively exercised at time course of being artificially inoculated by* G. boninense *and collected at week 1, 4, 8, and 12 after inoculation (WAI) according to the treatment application and inoculation method.

### 2.3. Biochemical Analysis Assays

The random excised tips of primary root tissues were gently washed with distilled water, wrapped with aluminum foil, and maintained in liquid nitrogen. After reaching the laboratory, the root tissues were weighed, before being frozen, and stored at −80°C for further analysis. The activity of four enzymes *β*-1,3-glucanase, chitinase, PAL, and POX was measured quantitatively using UV-1700 Pharmaspec, UV-VIS Spectrophotometer, Shimadzu Corporation, Japan.

### 2.4. *β*-1,3-Glucanase Activity

The activity of *β*-1,3-glucanase was determined according to the Nelson Somogyi method [17, 18], with slight modifications. The *β* −1,3-glucanase was assayed by measuring the rate of reducing sugar production with laminarin (Sigma Aldrich) as the substrate. Glucose was used as the standard and the activity was expressed as U mL^−1^ in unit^−1^ g tissue.

### 2.5. Chitinase Activity

The activity of chitinase was determined according to Tonon et al. with slight modifications [19]. Chitinase activity was assayed by measuring the rate of N-acetylglucosamine production using chitin (Sigma Aldrich) as the substrate. Chitinase activity was expressed as U mL^−1^ with N-acetylglucosamine as the standard.

### 2.6. Phenylalanine Ammonia-Lyase Activity

The activity of PAL was determined following the method of Chmielowska et al. with a slight modification [20]. The PAL activity was expressed in enzyme units (U) defined as *μ*mol of* trans*-cinnamic acid formed per minute, per mL of solution.

### 2.7. Peroxidase Activity

Peroxidase (POX) activity was measured following the modifications applied to the protocol of Chmielowska et al. [20]. The blank consisted of reaction mixture without enzyme extract. The POX activity was expressed in enzyme units (U) defined as *μ*mol of DAB oxidized per minute, per mL of solution.

### 2.8. Data Analysis

Data were compared by the analysis of variance (ANOVA) using SAS 9.3. When significant, means were compared by Duncan's Multiple Range Test (DMRT) at the 0.05 significance level. A *t*-test was performed for each treatment to compare infected and uninfected seedlings for plant biomass and for content in roots. All the assays were performed in triplicate using primary roots of noninoculated (healthy) and inoculated seedlings.

### 2.9. Metabolite Compounds Profiling

Based on the existence of diseases incidence responses, samples with the same treatments used for biochemical analysis were selected for this part of experiment. Aliquots of 500 mg oil palm leaf powder were extracted with 1.5 ml of 99.875% methanol acidified with formic acid 0.125%. The extracts were solicited, centrifuged, and filtered through a 0.2 *μ*m polytetrafluoroethylene (PTFE) filter. For each accession, two biological replicates were prepared, resulting in total of 40 extracts. To check the technical variation, including extraction, sample analysis, and data processing, quality control data were prepared by pooling leaf material of several randomly chosen accessions, extracted using the same procedure and injected after every 10 accession sample extract. All extracts were analyzed using reversed phase liquid chromatography with Mass-Spec (LC-MS) system, using C-18 reversed phase chromatography and negative electrospray ionization. About five *μ*m of the extract was injected and separated using a binary gradient of ultrapure water and acetonitrile, both acidified with 0.1% formic acid, with flow rate of 0.19 mL/min.

## 3. Results

### 3.1. Biochemical Analysis Assays

#### 3.1.1. Glucanase Activity

**Figure 1:**
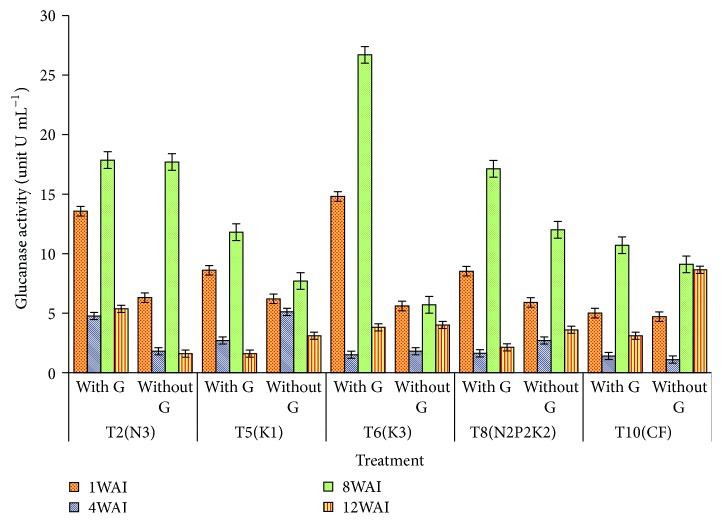
Effect of different levels of N, P, and K on glucanase activity in oil palm roots. Note. Values are the means of three replicates. Error bars represent the standard error. WAI: week after inoculation.

Glucanase enzyme activity showed high significant difference (*p* < 0.05) between the* Ganoderma* infected seedlings and control from week 1 to week 12 with *t*-value of 10.43, 6.74, 11.06, and 8.66 U mL^−1^ at 1 to 12 WAI, respectively (Table 3). The relatively low content of the uninfected seedlings is a reflection of the relatively low resistance towards* G. boninense* in the nutrient level studied. The time of defense related response induction was rapid, within week 1 and week 8, for *β*-1,3-glucanase in all the treatments (Table 3). Significant increase (*p* < 0.001) in glucanase activity on* G. boninense*-RWB seedlings was detected in most of the treatments at one WAI. However, T6 and T2 recorded great differences between control and +G inoculated oil palm seedlings while T5, T8, and T10 recorded slight changes among the treatments. By week 8, glucanase activity of T6 was dramatically higher, about 5-fold higher than untreated seedlings (Figure 1), as a sign of activated glucanase enzyme activity. However, *β*-1,3-glucanases are induced not only by pathogen infection, but also by other factors. Except for T10, at 12 WAI, the glucanase activity of all* G. boninense* seedlings of T2, T5, T6, and T8 was decreased gradually compared to the other treatments. However, result of OD recorded at 1, 8, and 12 WAI for glucanase activity of the* G. boninense*-RWB seedlings was notably higher than the control.

*Chitinase Activity*. A highly significant difference was found between the chitinase* Ganoderma* treatments and control from week 1 to week 12 with *t*-value of 11.54, 5.89, 8.34, and 5.16 U mL^−1^ for week 1 to week 12, respectively (Table 3). Although the level of chitinase increased in the* Ganoderma* infected seedlings during the study duration, it appeared that it was not sufficiently high, had not reached effective levels, or was too late to stop the fungal from growth.

**Figure 2:**
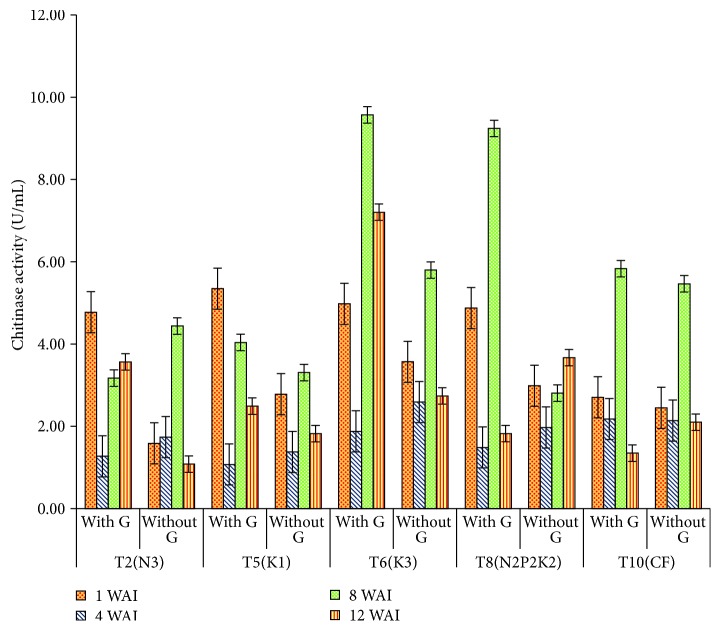
Effect of different levels of N, P_2_O_5_, and K_2_O on chitinase activity in oil palm roots. Note. Values are the means of three replicates. Error bars represent the standard error. WAI: week after inoculation.

Significant differences were observed at 8 to 12 WAI with T6 and T8 recording markedly higher enzyme activities compared to other treatments. Relatively low levels of chitinase were detected initially at 4 WAI for all different treatments and only began to produce higher levels of the enzyme at week 8 in both T6 and T8 and at week 12 in T6 when they were subjected to* Ganoderma* attack. Although, relatively high levels of chitinase were observed in most treatments at 1 WAIT, it does not seem to be vital for plants at the initial time period of treatment. The present results indicated that time of defense related response induction was at weeks 8 and 12 in T6 and at week 8 in almost all the treatments except T2 and T5. Treatments T6 and T8 at 8 WAI recorded the highest values of the chitinase activity. However, T2 recorded lower chitinase activity for +G inoculated seedlings treatments when compared to T6 and T8 (Figure 2). The low chitinase activity in T2 may be the cause of defense failure in the event of* Ganoderma *infection. All uninoculated seedlings from the uninoculated treatment, recorded lesser chitinase activity as compared to* Ganoderma*-inoculated treatment, which revealed a positive relationship at *p* < 0.05 between chitinase activity and* G. boninense* infected seedlings.

**Figure 3:**
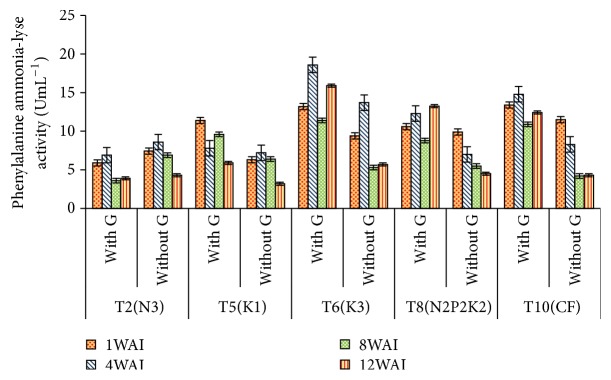
Effect of different levels of N, P_2_O_5_, and K_2_O on PAL activity in oil palm roots. Note. Values are the means of three replicates. Error bars represent the standard error. WAI: week after inoculation.

*Phenylalanine Ammonia-Lyase Activity*. High significant difference was found between the PAL* Ganoderma t*reatment and control from week 1 to week 12 with *t*-value of 13.87, 11.58, 11.72, and 9.47 for week 1 to week 12, respectively (Table 3). The PAL activity measured in the roots of infected palms revealed highly significant differences among treatments (*p* < 0.0001). The highest activity was observed in the infected roots of T6, followed by T8 and T10 with no significant difference between them, while the lowest was detected in T2. This could indicate that at this stage more phenolics including lignin were synthesized in T6, T8, and T10 and are more tolerant compared to T2 (Figure 3).

**Figure 4:**
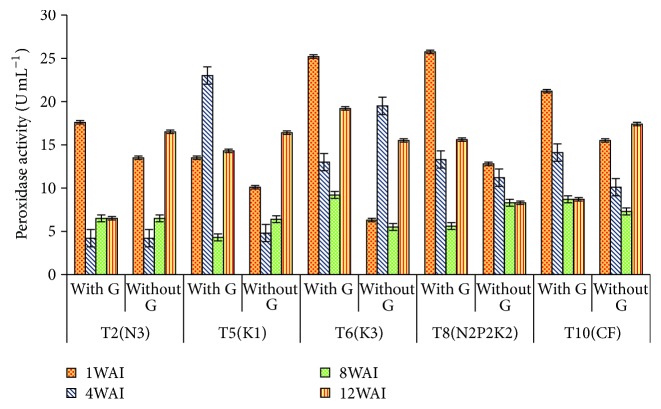
Effect of different levels of N, P_2_O_5_, and K_2_O on POX activity in oil palm roots. Note. Values are the means of three replicates. Error bars represent the standard error. WAI: week after inoculation.

*Peroxidase Activity*. High significant difference was found between the* Ganoderma *treatment and control from week 1 to week 12 with *t*-value of 12.82, 6.60, 16.74, and 15.74, respectively. Higher POX was expressed in T8 at 1 WAI, but T6 showed higher POX activities from 8 WAI to 12 WAI. The lowest POX activity in the root was in sample T2 (Table 3). The results obtained have shown that T6 significantly gave high value for enzyme POX activity. However, at 1 WAI, T8 was the highest followed by T10, an indication of a fast and rapid induction of POX at the beginning of infection. However, at 4, 8, and 12 WAI, T6 gave the highest POX activity and was significantly higher in all infected root tissues. At 8 WAI, all POX activities were not significantly different among the* Ganoderma* infected root tissues for POX enzymes. Glucanase, POX (weeks 1 and 4), and PAL activities were stimulated by the* Ganoderma* infection within 1 week, recurring at week 8, and chitinase within week 8 after treatment application (Figure 4).

*Metabolite Compounds of Uninfected and Infected Oil Palm Seedlings*. Several metabolite compounds were detected in uninfected and infected seedlings treated with different levels of N, P, and K fertilizer. However, only known compounds are listed here to study more details and find interaction between effect of pathogen and fertilizer (Table 4). There was a big difference observed in the number of released metabolite compounds between uninfected/infected samples with different N, P, and K treatments most probably due to alteration in antioxidant enzyme activities. For instance, forty compounds were obtained from uninfected sample T1 with basal fertilizer (BF) and low N fertilizer, while only three compounds were extracted from the same fertilized sample T1 infected with* Ganoderma*. One and five compounds were observed, respectively, from uninfected and infected sample T2 with BF and high N fertilizer. One compound was obtained from uninfected sample T3 with basic fertilizer (BF) and low P fertilizer, while 18 compounds were extracted from the same fertilized sample T3 infected with* Ganoderma*. One and 13 compounds were observed, respectively, from uninfected and infected sample T4 with BF and high P fertilizer. Two compounds were obtained from uninfected sample T5 with basic fertilizer (BF) and low K fertilizer, while nine compounds were extracted from the same fertilized sample T5 infected with* Ganoderma.* Four and two compounds were observed, respectively, from uninfected and infected sample T6 with BF and high K fertilizer.

Three compounds were obtained from uninfected sample T7 with basic fertilizer (BF) treated with N1P1 K1 (N low, P low, K low), while ten compounds were extracted from the same fertilized sample T7 infected with* Ganoderma*. Two compounds were obtained from uninfected sample T8 with basal fertilizer (BF) treated with N2P2 K2 (N optimum, P optimum, K optimum), while five compounds were extracted from the same fertilized sample T8 infected with* Ganoderma*. Thirty-one compounds were obtained from uninfected sample T9 with basic fertilizer (BF) treated with N3P3 K3 (N high, P high, K high), whereas unknown compounds were identified from the same fertilized sample T9 infected with* Ganoderma*. Finally, twenty-six compounds were obtained from uninfected sample T10 with basic fertilizer (BF) and 14 compounds were extracted from the same fertilized sample T10 infected with* Ganoderma. *The number of detected known metabolite compounds differs among infected and uninfected samples. For instance, the number of known compounds extracted from uninfected sample T1, T6, T9, and T10 was higher than that from infected ones, while the number of compounds extracted from infected samples T2, T3, T4, T5, T7, T8 was higher than that from uninfected ones.

**Figure 5:**
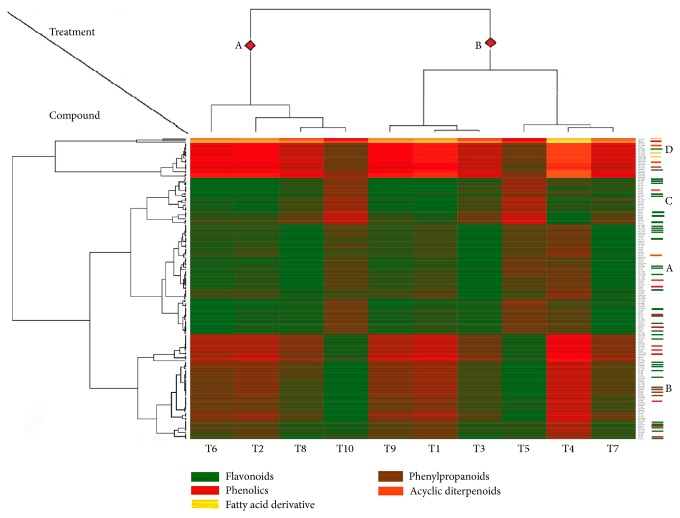
Heatmap of 190 metabolite compounds in 10 types of NPK treatment oil palm seedling. A color matrix represents the mean values of the metabolite compounds in two biological replicates of oil palm. Characterization of the underlying metabolites is presented in Table 4.

**Figure 6:**
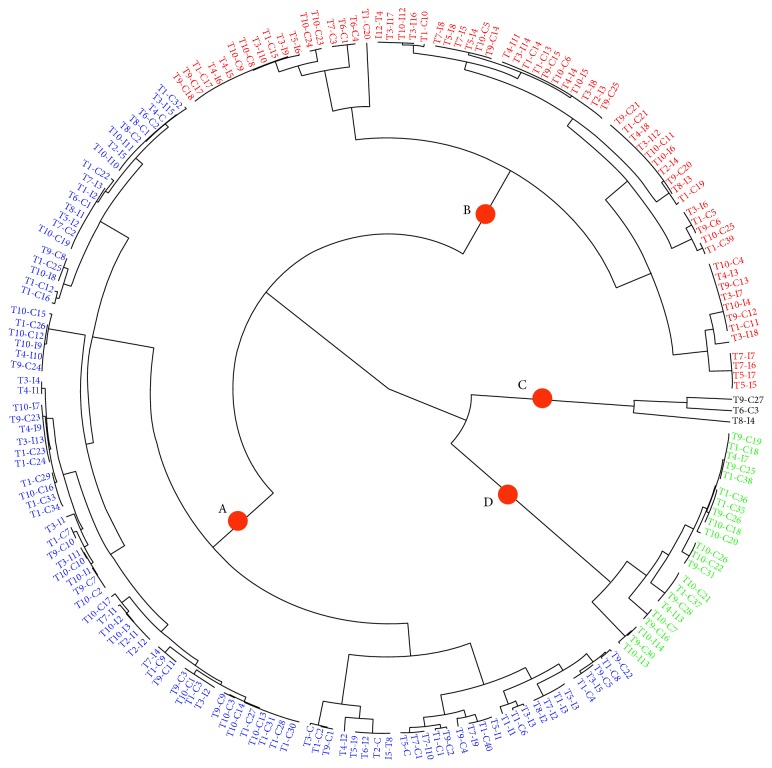
Fan plot showing all metabolite compounds of oil palm in both forms of control (−G) and inoculated with* Ganoderma* (+G), under different NPK treatments. Four different groups A, B, C, and D represent the* m/z* mean values of the metabolite compounds including flavonoids, phenylpropanoids, acyclic diterpenoids, and fatty acid derivative.

**Figure 7:**
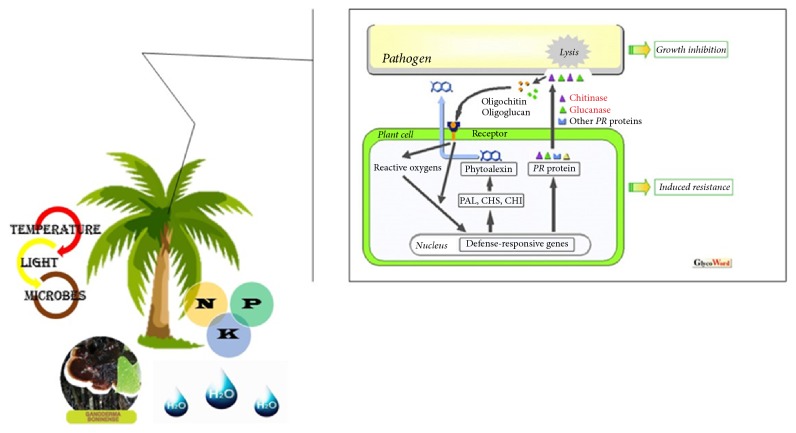
Schematic model of oil palm,* Ganoderma boninense*, fertilizer regime interaction focusing on the role of plant lytic enzymes (Glycoforum: http://www.glycoforum.gr.jp). This schematic model indicates the contribution of PR proteins in response to macronutrients nutrition in oil palm with* Ganoderma* infection. Plants produce *β*-1,3-glucanases, chitinase, POX, and PAL during the process of pathogenesis for self- defense, and pathogens struggle against them. Application of NPK nutrients, especially K fertilizing, might either act as elicitors triggering the production of glucanase, chitinase, POX, and PAL activities or be involved in secondary metabolite profiling associated with induced resistance.

The dendrogram and fan plot produced by hierarchal cluster analysis (HCA) of the different fertilized oil palm seedlings, based on a common molecular mass deduced from the recorded *m*/*z* value and similar retention time (RT) and peak shape (Figures 5 and 6), provided a more detailed view on the relationships between treatments. The HCA distinguished two major clusters for treatments denoted as A and B. Cluster A consist of [T2, T6, T8, T10] with the higher enzyme activities among other treatments. Cluster B contained two subgroups: one consisting of [T1, T3, T9] and a second one consisting of [T4, T5, T7]. The HCA of the set of 190 metabolite compounds revealed the presence of several metabolite groups, arbitrarily denoted with A to D, characterized by their particular expression pattern across the ten treatments (Figure 6). Although there is no direct evidence to show the extent of plant enzymes contributing to the resistance at the infection site because they are interacted by various factors and encoded by multigene family, a previous considerable research provided corroborative evidence that plant chitinase and *β*-1,3-glucanases in plant self-defense mechanism (Figure 7) illustrate how optimum NPK nutrients might either mutate the production of glucanase, chitinase, POX, and PAL activities or be involved in secondary metabolite profiling for basal resistance in oil palm.

## 4. Discussion

Induction of *β*-1,3-glucanases and other PR proteins in the plant can also occur due to some components of pathogens or degraded components of pathogens. These elicitors may be components of the cell surface of the pathogen that are released by host enzymes, including fungal *β*-glucan, chitin, chitosan, glycoproteins, and N-acetyl- chitooligosaccharides [21, 22]. They may also be synthesized and released by the pathogen after it enters the host in response to host signals. In view of the obtained results from chitinase assay in this study, it appears likely that chitinase plays a role in the interaction between* Ganoderma* and nutrition of the oil palm seedlings. As pointed out by other researchers [23, 24], the early and massive induction of chitinase activity is generally related to the increased resistance of plant tissues to infection by pathogens. However, at week 12, the chitinases activity was significantly decreased with the increase in time over the study period, an indication that the enzyme activities were short-lasting. This has been supported previously by the fact that defense mechanisms are often activated late in the infection process when the pathogen had colonized the tissue and the plant can no longer benefit from these mechanisms [25].

It was reported that after a plant was challenged by potentially pathogenic microorganisms, the plant will respond by effecting changes in the composition and physical properties of its cell walls [26]. Firstly, they can degrade the cell wall of the pathogen or disrupt its deposition, eventually contributing to the pathogen death. Secondly, they can release cell wall fragments that act as elicitors of active host defense response. In addition, the plant will produce secondary metabolites that serve to isolate and limit the spread of the invading pathogen and also limit necrotic lesions at the site of the invasion. These responses are collectively known as hypersensitive reaction. Following infection, PR proteins accumulate in leaves and other organs, where they may comprise more than 10% of the total soluble protein. It was reported that there is a close relationship between glucanase and chitinase activity because it has been proposed that glucanase and chitinase would act synergistically together to affect or inhibit fungal growth either by lysis of the hyphal tips [26] or by interfering with the correct balance between fungal cell wall synthesis and wall hydrolysis during cell wall extension of the hyphal tip [27, 28].

Although the level of PR proteins increased in the* G. boninense *infected seedlings for the trial duration period, it appears that they are not sufficient or reach effective levels too late to stop the fungal from growth. This was supported by Van Loon who reported that defense mechanisms are often activated late in the infection process when the pathogen had colonized the tissue and the plant can no longer benefit from these mechanisms [25]. Low level of chitinase activity may also be the factor of high susceptibility among oil palm seedlings to* G. boninense* infection. Other than that, low level of chitinase activity may lower the hydrolysis effect of glucanase activity since both enzymes work synergistically [26]. With the rapid advancement in genetic transformation, oil palm seedlings resistance to* Ganoderma* infection might be improved by regulation of chitinase gene expression and other genes controlling host resistance [29]. As mention earlier, PAL catalyzes the first committed step in phenylpropanoid metabolism which is vital for the synthesis of phenolic compounds including lignin [30]. Most evidence suggests that PAL activity is correlated not only with lignin biosynthesis, but also with the production of salicylic acid precursors. Salicylic acid is reported to play a central role in genetically determined plant disease resistance [31]. Apart from that, the POX activity is not always associated with lignification [32]. The POXs are mainly involved in hydrogen peroxide (H_2_O_2_) scavenging to alleviate oxidative stress caused by reactive oxygen species [20], thereby playing a detoxifying role. Since they are also implicated in tissue growth and differentiation in plants, expression of peroxidases in roots might indicate simple root differentiation activity and not necessarily lignin biosynthesis [33].

As has been known, POXs are key enzymes in the cell wall-building process, and it has been suggested that extracellular or wall-bound peroxidases would enhance plant resistance by the construction of a cell wall barrier that may impede pathogen ingression and spread [34]. Peroxidases are also involved in HR and for the polymerization of cell wall components such as lignin, suberin, and extensin, leading to the formation of barriers for infecting pathogens [35]. Even though many peroxidases are found in most plant species and are expressed constitutively, some isozymes appear to be inducible upon pathogen infection.

The application of N, P, and K nutrients as observed here may play important role in directly stimulating the inducible compounds in oil palm seedlings. Although this study has also shown that macronutrient application has not stopped the progress of* G. boninense*, the infection was delayed. The occurrence of induced resistance subsequently implied enhanced tolerance of oil palm seedlings towards development of* G. boninense* and delayed the onset of symptoms. Effect of different nutrients doses was obviously observed among results of secondary metabolite compounds. For instance, only a few but important number of metabolite compounds observed in sample T6 leads to the highest activities of four antioxidant enzymes activities. That shows the significant importance of K fertilizing for oil palm trees among other fertilizers. Although highest N fertilizing of sample T2 led plants to produce different metabolite compounds and consequently caused the highest activities of glucanase activities, it failed for chitinase activities among other treatments, which is most probably due to lack of enough K. Sample T8 with optimum levels of NPK fertilizing and extracted different metabolite compounds showed the highest activities for all antioxidant enzyme activities among other treatments. Samples T1, T3, T4, T5, T7, and T9 that were treated with different levels of fertilizing, produced variety of metabolite compounds mostly in control type (−G), which were not effective enough for plants after inoculation with* Ganoderma*. Extracted metabolite compound as well as results of antioxidant enzyme activities of samples T10 with basic fertilizer (BF) and T8 with optimum level of fertilizers also confirmed the importance of using balanced NPK for oil palm trees. It is difficult to determine exact suppression mechanism with the application of macronutrients since other complex interactions in the soil-plant-pathogens exist. Moreover,* G. boninense* is notoriously adaptable, and many plant and nutrient factors may interact to contribute to different susceptibility to the oil palm seedlings. On the other hand, role of most metabolite compounds presented in oil palm seedlings under different N, P, and K treatments against* Ganoderma* attack has not been clear yet, though many of them have been identified in other plants as useful medicinal compounds for human health. Identifying and categorizing these compounds and their effective relation in oil palm seedlings against* Ganoderma* attack may be used as new markers for the early detection of disease. At the next step, besides explaining the role of micronutrients in antioxidant enzymes activities, identifying the role of extracted secondary metabolite compounds may help to formulate improved fertilizers for oil palm trees. It was revealed that susceptibility may be associated with cell maintenance and development, genes involved in the biosynthesis of lignin and phenolics and genes implicated in oxidative burst, programmed cell death, or hypersensitive responses [36].

The* G. boninense* disturbs the lignin and other structural components of the cell wall during infection using its enzyme activities [37, 38]. Lignin protects both cellulose and hemicellulose components from fungus enzymatic activities through chemical bonds.* Ganoderma* attacks the oil palm, and due to the lack of sufficient lignin, the permeability of cell wall increased. Lignin plays an important role in protecting plants from microbial degradation and decay. Also, it limits the plant's biomass conversion to biofuels. Besides all the positive effects of lignin in plant cell wall like protection of plant against several stress conditions, for the purpose of some investigation, existence of lignin will be undesirable trait. Lignin has an important role in plant defense against pathogen invasion. Lignification is a mechanism for resistance in plants. After pathogen invades the plant, lignin or lignin-like phenolic compound accumulation was shown to occur in a variety of plant-microbe interactions during the plant defense responses. Endogenous enzymes chitinase, *β*-1,3-glucanases, and lignin content in plant leaves can be used as biochemical markers for identifying plant varieties resistant to fungal infection or other biotic and abiotic factors. Also by transferring pathogenesis-related (PR) proteins, such as chitinases, *β*-1,3-glucanases genes can induce resistance in plants to various pathogens.

During the degradation of cell wall in oil palm, an array of cell wall degrading enzymes are expected to be released by* Ganoderma *to ensure successful colonization and degradation of the intact root tissues. The defense mechanism fails if a virulent pathogen, such as* G. boninense*, avoids triggering, suppresses resistance reaction, or evades the effects of activated defense in oil palm seedlings. Consequently, the infection becomes apparent. Researchers indicated that a disease can be reduced when defense mechanisms are triggered by a stimulus prior to infection by a plant pathogen [25, 39]. Hence, lignin is an obstacle point to protect the inner cell wall against fungus infections [40]. It has been reported that decreasing the susceptibility of plants against fungus has been achieved by increasing lignin in plant cell wall [41]. Therefore, it seems that using these mechanisms may increase* G. boninense* tolerance in oil palm. Lignocelluloses refer to plant cell in which lignin is associated closely with cellulose and hemicelluloses of cell wall. Lignin acts as a physical barrier to pathogen attack and, in the xylem tissue of plants, provides a water impervious seal throughout cell walls. Lignin forms chemical bonds directly against enzymatic attack of pathogens and protects more amenable hemicelluloses and cellulose [42]. The polymeric lignin structure involves at least three building units in monomeric form. The term “lignin” refers to a big group of polymers involved in oxidative combinatory of 4-hydroxyphenylpropanoids [43]. Hydroxycinnamyl alcohols, sinapyl alcohol, coniferyl alcohol, and a small amount of p-coumaryl alcohol are involved in the formation of the main composition of the lignin building [40].

Mineral nutrition also affects the formation of mechanical barriers in plant tissue. As leaves age, the accumulation of silicon (Si) in the cell walls helps form a protective physical barrier to fungal penetration [44, 45]. Excessively high N levels lower the Si content and increase susceptibility to fungal diseases.

The secondary cell wall between the middle lamella and the plasma membrane is deposited with a large amount of lignin. Although the composition of lignin, pectin, hemicelluloses, and cellulose microfibril leads to the production of the impervious and rigid secondary cell wall in plants, lignin is the most effective factor on the secondary cell wall structure. However,* Ganoderma* needs other sources of food, such as cellulose and pectin rather than lignin, to be able to break down plant. Notwithstanding various factors to increase lignin in plant cell wall, it was reported that the most effective factors of lignin biosynthesis are abiotic and biotic stresses [46]. The* Ganoderma boninense* obtains its required energy for causing the disease from lignin degradation of cell wall, although Paterson reported that pectin and starch also play a light role in surviving fungi [42]. When* Ganoderma* attacks the plants, spore of fungi in favorable condition starts to develop its new cells, and then fungi cells become hyphae that later create mycelium. Mycelium of fungi forms a lump which grows out of the soil when mycelium penetrates into the oil palm cells; it uses the lignin of the cell wall and continues to destruct the plant. When* G. boninense* attacks the oil palm, it tries to reach the lignin components of the cell wall; this period of time in different plants may vary based on cell wall thickness.

In a research on the BSR of oil palm, infection symptoms appeared on the roots of inoculated plants throughout 5 months [47]. It was shown that penetration of fungus mycelium into the cell wall of oil palm root surface occurred in both epidermis and exodermises layers; this engagement between fungus and root surface can be limited to the primary contact point or cover root completely [37]. It can be understood that the main and the most important step in controlling* Ganoderma *disease is related to the cell wall thickness, particularly in the first layer. By this way, the fungi mycelium contact with the middle lamella of the plant cell wall will be limited. Thus, the BSR disease rate of oil palm will reduce. It was revealed that a* Ganoderma* colonization may occur along with unwounded root parts and then may develop mostly through the cortex part of the inner cell wall [37].

## 5. Conclusion

This study suggests the involvement of PR proteins in response to macronutrients nutrition in oil palm with and without* Ganoderma* infection. The observed trend of changes in the infected and uninfected plants was a slightly higher activity for *β*-1,3-glucanases, chitinase, POX, and PAL during the process of pathogenesis. The PR proteins gave positive response to the interaction between oil palm seedlings and* Ganoderma* infection. Although the response was activated systematically, they were short-lasting as the changes in enzymes activities appeared before the occurrence of visible symptoms. These enzymatic reactions may be useful as early markers of stress condition in oil palm to* Ganoderma* infection. Though direct evidence for the causal role of these enzymes was lacking, this study has proved that the accumulation of PR in oil palm seedlings infected by* Ganoderma* may be related to systemic acquired resistance (SAR). This is because there were some changes in the PR proteins that can be detected in the roots. This indicated that the increase in the PR proteins was a plant response to infection, not simply a contribution of the hyphal enzymes per se. On the other hand, the metabolite profiling of oil palm offers an improvement to the comprehending of the biosynthetic pathways responses to environmental changes and acts as the platform for further exploration and identification of different metabolite markers for BSR disease detection.

However, whether or not the N, P, and K macronutrients application resulted in PR proteins induction, which plays a direct role in the disease resistance, remains unclear and needs to be further investigated. Application of NPK nutrients, especially K fertilizing, might either act as elicitor triggering the production of glucanase, chitinase, POX, and PAL activities or be involved in secondary metabolite profiling associated with induced resistance. Many plant enzymes are involved in defenses reactions against plant pathogens. The results of this study could be useful for developing new strategies with proper nutrition, which may decrease* Ganoderma* diffusion and its growth rate in peat soil.

## Figures and Tables

**Table 1:** Fertilizer treatment levels for healthy and (+G) seedlings inoculated with *G. boninense*.

T1 = seedling treated with N1^*∗*^ (N low) + BF
T2 = seedling treated with N3^*∗*^ (N high) + BF
T3 = seedling treated with P1^*∗∗*^ (P low) + BF
T4 = seedling treated with P3^*∗∗*^ (P high) + BF
T5 = seedling treated with K1^*∗∗∗*^ (K low) + BF
T6 = seedling treated with K3^*∗∗∗*^ (K high) + BF
T7 = seedling treated with N1P1K1 (N low, P low, K low) + BF
T8 = seedling treated with N2P2K2 (N optimum, P optimum, K optimum) + BF
T9 = seedling treated with N3P3K3 (N high, P high, K high) + BF
T10 = seedling treated with compound fertilizer (CF)

*Note.* ^*∗*^P and K optimum, ^*∗∗*^N and K optimum, ^*∗∗∗*^N and P optimum. All treatments with BF = basic fertilizer 2% MgO (source from kieserite) + micronutrients (TE). In the common commercial name (CF), where N stands for % N (12%), P % for P_2_O_5_ (12%), K for % K_2_O (17%), and Mg for % MgO (2%) + TE.

**Table 2:** Fertilizer and rates applied in the nursery trials.

Month		Urea			TSP			MOP	
	N1	N2	N3	P1	P2	P3	K1	K2	K3
	g/palm								

4	0.69	0.99	1.29	0.693	0.99	1.29	1.15	1.64	2.13
5	0.80	1.14	1.48	0.798	1.14	1.48	1.34	1.92	2.50
6	0.88	1.26	1.64	0.882	1.26	1.64	1.57	2.24	2.91
7	0.97	1.38	1.79	0.966	1.38	1.79	1.74	2.48	3.22
8	1.03	1.47	1.91	1.029	1.47	1.91	1.90	2.72	3.54
9	1.11	1.59	2.07	1.113	1.59	2.07	2.07	2.96	3.85
10	1.20	1.71	2.22	1.197	1.71	2.22	2.24	3.20	4.16
11	1.26	1.80	2.34	1.26	1.8	2.34	2.38	3.40	4.42
12	1.34	1.92	2.50	1.344	1.92	2.50	2.49	3.56	4.63
13	1.41	2.01	2.61	1.407	2.01	2.61	2.63	3.76	4.89
14	1.47	2.10	2.73	1.47	2.1	2.73	2.74	3.92	5.10

Total	12.16	17.37	22.58	12.16	17.37	22.58	22.26	31.80	41.34

*Note.* Application of fertilizer in eleven months of experiment for growth analysis of oil palm seedlings. Amount of fertilizer needed for 1 replication for each level (g); TSP: triple superphosphate, MOP: muriate of potash. Level N1, P1, K1 (30%) less than N2, P2, and K2. Level N3, P3, and K3 (30%) more than N2, P2, and K2.

**Table 3:** Effect of N, P, and K nutrition and different enzyme activities of healthy and *Ganoderma* infected oil palm seedlings.

Week after inoculation	*Ganoderma* infected seedlings (UmL^−1^)	Control (Um L^−1^)	*t* value	T2	T5	T6	T8	T10
	Glucanase activity			Effect of N, P_2_O_5_, and K_2_O nutrition on glucanase activity				

1WAI	10.097 ± 0.96	5.74 ± 0.33	10.43s^*∗*^	9.93^a^	7.4^b^	10.2^a^	7.2^b^	4.9^c^
4WAI	2.397 ± 0.35	2.5 ± 0.47	6.74s^*∗*^	3.3^ab^	3.90^a^	1.65^c^	2.16^bc^	1.25^c^
8WAI	16.837 ± 1.5	10.44 ± 1.15	11.06s^*∗*^	17.78^a^	9.75^d^	16.2^b^	14.56^c^	9.9^d^
12WAI	3.197 ± 0.36	4.188 ± 0.69	8.66s^*∗*^	3.48^bc^	2.35^c^	3.9^b^	2.9^bc^	5.87^a^

	Chitinase activity			Effect of N, P_2_O_5_, and K_2_O nutrition on chitinase activity				

1WAI	4.53 ±0.39	2.67 ± 0.46	11.54s^*∗*^	3.18	4.07	4.27	3.93	2.57
4WAI	1.57±0.26	1.96 ± 0.33	5.89s^*∗*^	1.50	1.22	2.23	1.73	2.16
8WAI	6.37± 0.76	4.36 ± 0.52	8.34s^**∗**^	3.80^c^	3.67^c^	3.68^a^	6.03^ab^	5.65^b^
12WAI	3.28± 0.63	2.28 ± 0.39	5.16s^**∗**^	2.32^b^	2.16^b^	4.97^a^	2.75^b^	1.73^b^

	PAL activity			Effect of N, P_2_O_5_, and K_2_O nutrition on PAL activity				

1WAI	10.9 ±0.89	8.90 ± 0.64	13.87s^*∗*^	6.67^c^	8.85^bc^	11.30^ab^	10.25^ab^	12.45^a^
4WAI	12.08 ± 1.27	8.96 ± 0.77	11.58s^*∗*^	7.75^c^	7.50^c^	16.15^a^	9.65^bc^	11.55^b^
8WAI	8.86 ± 0.90	5.66 ±0.48	11.72s^*∗*^	5.25^b^	8.00^ab^	8.35^a^	7.15^ab^	7.55^ab^
12WAI	10.28 ± 1.33	4.4 ± 0.46	9.47s^*∗*^	4.10^b^	4.55^b^	10.81^a^	8.88^a^	8.37^a^

	POX activity			Effect of N, P_2_O_5_, and K_2_O nutrition and BSR on POX Activity				

1WAI	20.65 ± 1.42	11.64 ± .91	12.82s^**∗**^	15.55^b^	11.80^c^	15.75^b^	19.26^a^	18.35^ab^
4WAI	13.52 ± 1.74	9.96 ± 1.51	6.60s^**∗**^	4.20^c^	13.90^ab^	15.25^a^	12.25^b^	12.1^b^
8WAI	6.86 ± 0.85	6.8 ±0.40	16.74s^**∗**^	6.50^a^	5.35^a^	7.35^a^	6.95^a^	8.00^a^
12WAI	12.86 ± 1.43	14.82 ± .94	15.74s^**∗**^	11.50^c^	15.35^ab^	17.35^a^	11.95^bc^	13.05^bc^

*Note.* values are the means of three replicates;  s^**∗**^ indicates highly significant according to *T*-test at *α*′ = 0.05. WAI: week after inoculation; all the *t*-values are highly significant at 0.001%. Means with the same letter at a given parameter are not significantly different according to Duncan's Multiple Range Test (DMRT) at *α* = 0.05; values are the means of three replicates ± standard error. Treatments: T2 = N3+ BF + *Ganoderma*; T5 = K1 + BF + *Ganoderma*; T6 = K3 + BF + *Ganoderma*; T8 = N2P2K2 + BF + *Ganoderma*; T10 = control (compound fertilizer) + BF + *Ganoderma.*

**Table 4:** Mass spectrometry for each compound and its molecular structure observed in different N, P, K combination of *Ganoderma* inoculated (+) and uninoculated oil palm seedlings.

Sample	*Ganoderma*	RT	*m/z*	Mass	Name	Molecular formula
T1	(−)	0.87	175.119	174.112	Arginine	C6 H14 N4 O2
T1	(−)	0.92	104.107	103.1	2-Amino-3-methyl-1-butanol	C5 H13 N O
T1	(−)	1	293.066	254.101	Dyphylline	C10 H14 N4 O4
T1	(−)	1.01	203.054	180.065	Theobromine	C7 H8 N4 O2
T1	(−)	1.02	435.062	180.065	4-Hydroxylevamisole glucuronide	C17 H20 N2 O7 S
T1	(−)	1.13	220.134	180.065	Meperidinic acid	C13 H17 N O2
T1	(−)	1.2	268.155	180.065	Metoprolol acid	C14 H21 N O4
T1	(−)	1.24	206.119	180.065	Normeperidinic acid	C12 H15 N O2
T1	(−)	1.25	282.171	180.065	Benzenepropanoic acid, 4-[2-hydroxy-3-	C15 H23 N O4
					[(1-methylethyl)amino]propoxy]-(9CI) ASL 8123	
T1	(−)	1.93	417.18	180.065	S-adenosylmethionine	C15 H23 N6 O5 S
T1	(−)	1.96	362.209	180.065	Phe Pro Val	C19 H27 N3 O4
T1	(−)	2.18	234.15	180.065	Normeperidine	C14 H19 N O2
T1	(−)	2.23	409.185	180.065	Asn Met Gln	C14 H25 N5 O6 S
T1	(−)	2.31`	409.185	180.065	Asn Met Gln	C14 H25 N5 O6 S
T1	(−)	2.89	480.226	180.065	Gln Phe Trp	C25 H29 N5 O5
T1	(−)	3.17	236.166	180.065	5,7,9,11,13-tetradecapentaenoic acid	C14 H18 O2
T1	(-)	3.23	480.224	180.065	Gln Phe Trp	C25 H29 N5 O5
T1	(−)	3.47	601.154	180.065	Rhoifolin	C27 H30 O14
T1	(−)	4.71	393.19	180.065	Trp Gly Asn	C17 H21 N5 O5
T1	(−)	4.89	541.263	180.065	17-Alpha, 21-dihydroxy-11,20-dioxo-5-beta-pregnan-	C27 H40 O11
					3-alpha-yl-beta-d-glucuronide	
T1	(−)	5.26	395.205	180.065	Tyr Gly Arg	C17 H26 N6 O5
T1	(−)	9.75	246.243	180.065	4,8-Dimethyl-dodecanoic acid	C14 H28 O2
T1	(−)	12.1	274.275	180.065	C16 sphinganine	C16 H35 N O2
T1	(−)	12.1	274.275	180.065	C16 sphinganine	C16 H35 N O2
T1	(−)	12.2	230.249	180.065	Myristaldehyde	C14 H28 O
T1	(−)	12.2	318.301	180.065	Phytosphingosine	C18 H39 N O3
T1	(−)	12.2	288.291	180.065	C17 sphinganine	C17 H37 N O2
T1	(−)	12.4	288.291	180.065	C17 sphinganine	C17 H37 N O2
T1	(−)	12.6	272.259	180.065	2R-aminohexadecanoic acid	C16 H33 N O2
T1	(−)	12.8	288.291	180.065	C17 Sphinganine	C17 H37 N O2
T1	(−)	13.2	288.291	180.065	C17 Sphinganine	C17 H37 N O2
T1	(−)	13.3	244.265	180.065	9-Pentadecen-1-ol	C15 H30 O
T1	(−)	15	272.296	180.065	14-Methyl-8-hexadecen-1-ol	C17 H34 O
T1	(−)	15.5	272.296	180.065	14-Methyl-8-hexadecen-1-ol	C17 H34 O
T1	(−)	16.2	599.412	180.065	Mytiloxanthin	C40 H54 O4
T1	(−)	21.3	599.412	180.065	Mytiloxanthin	C40 H54 O4
T1	(−)	21.5	585.433	180.065	Myxol	C40 H56 O3
T1	(−)	21.5	601.427	180.065	Capsanthin 3,6-epoxide	C40 H56 O4
T1	(−)	21.8	429.374	180.065	25-Hydroxy-26,27-dimethylvitamin D3/	C29 H48 O2
					25-Hydroxy-26,27-dimethylcholecalciferol	
T1	(−)	22.1	167.01	180.065	6-Hydroxy-2-hexynoic acid	C6 H8 O3
T1	(+)	8.79	213.145	180.065	meglumine	C7 H17 N O5
T1	(+)	17.8	248.198	180.065	Leu Val	C11 H22 N2 O3
T1	(+)	17.8	192.135	180.065	Lupinine	C10 H19 N O
T2	(−)	21.92	125.039	248.064	Asp Asp	C8 H12 N2 O7
T2	(+)	1.06	300.148	299.14	Metoclopramide	C14 H22 Cl N3 O2
T2	(+)	1.17	300.148	299.141	Metoclopramide	C14 H22 Cl N3 O2
T2	(+)	2.28	409.187	408.18	Trp Phe Gly	C22 H24 N4 O4
T2	(+)	5.24	395.208	356.246	Dopexamine	C22 H32 N2 O2
T2	(+)	17.77	243.139	242.132	6E,8E,14E-hexadecatriene-10,12-diynoic acid	C16 H18 O2
T3	(−)	22.09	102.971	64.0082	Ethyl chloride	C2 H5 Cl
T3	(+)	0.26	257.975	219.011	4-Amino-3-(5-chlorothien-2-YL) Butanoic acid	C8 H10 Cl N O2 S
T3	(+)	0.97	293.066	254.103	Dyphylline	C10 H14 N4 O4
T3	(+)	0.98	219.028	180.065	Theobromine	C7 H8 N4 O2
T3	(+)	1	277.0914	254.102	Dyphylline	C10 H14 N4 O4
T3	(+)	1.01	203.055	180.065	Theobromine	C7 H8 N4 O2
T3	(+)	1.03	435.061	396.097	4-Hydroxylevamisole glucuronide	C17 H20 N2 O7 S
T3	(+)	2	362.244	344.21	Oxyphencyclimine	C20 H28 N2 O3
T3	(+)	2.27	409.187	408.18	Trp Phe Gly	C22 H24 N4 O4
T3	(+)	2.9	480.227	479.219	Gln Phe Trp	C25 H29 N5 O5
T3	(+)	3.24	480.226	479.219	Gln Phe Trp	C25 H29 N5 O5
T3	(+)	3.63	264.161	246.127	Santonin	C15 H18 O3
T3	(+)	5.26	395.207	377.174	Gln Lys Cys	C14 H27 N5 O5 S
T3	(+)	12.07	274.275	273.268	C16 Sphinganine	C16 H35 N O2
T3	(+)	12.49	409.16	370.197	Lys His Ser	C15 H26 N6 O5
T3	(+)	16	243.134	242.126	Hydroxyamobarbital	C11 H18 N2 O4
T3	(+)	18.2	419.243	418.236	Met Arg Leu	C17 H34 N6 O4 S
T3	(+)	18.22	419.279	418.272	Simvastatin	C25 H38 O5
T3	(+)	18.23	375.253	374.245	Digitoxigenin	C23 H34 O4
T4	(−)	16.01	243.133	242.126	Hydroxyamobarbital	C11 H18 N2 O4
T4	(+)	0.99	277.091	254.102	Dyphylline	C10 H14 N4 O4
T4	(+)	1.01	138.058	120.025	Sulfolane	C4 H8 O2 S
T4	(+)	1.95	362.243	344.209	Oxyphencyclimine	C20 H28 N2 O3
T4	(+)	2.25	409.186	408.178	Trp Phe Gly	C22 H24 N4 O4
T4	(+)	2.86	480.226	479.218	Gln Phe Trp	C25 H29 N5 O5
T4	(+)	3.2	480.226	479.219	Gln Phe Trp	C25 H29 N5 O5
T4	(+)	3.44	601.156	578.166	Rhoifolin	C27 H30 O14
T4	(+)	5.23	395.207	377.173	Gln Lys Cys	C14 H27 N5 O5 S
T4	(+)	12.06	274.275	273.268	C16 sphinganine	C16 H35 N O2
T4	(+)	12.18	318.3	317.292	Phytosphingosine	C18 H39 N O3
T4	(+)	12.48	409.163	386.174	Lys Cys His	C15 H26 N6 O4 S
T4	(+)	18.19	419.279	418.271	Simvastatin	C25 H38 O5
T4	(+)	21.49	585.433	584.426	Myxol	C40 H56 O3
T5	(-)	22.61	179.988	141.025	5-Acetyl-4-methylthiazole	C6 H7 N O S
T5	(−)	22.93	*―*	141.025	5-Acetyl-4-methylthiazole	C6 H7 N O S
T5	(+)	1.35	167.01	128.047	6-Hydroxy-2-hexynoic acid	C6 H8 O3
T5	(+)	17.76	248.196	230.162	Leu Val	C11 H22 N2 O3
T5	(+)	17.77	192.136	169.147	Lupinine	C10 H19 N O
T5	(+)	21.45	411.2875	410.2796	24,25-epoxy-1 alpha-hydroxy-22,22	C27 H38 O3
T5	(+)	21.46	343.2247	320.2348	(+/−)14,15-EpETrE	C20 H32 O3
T5	(+)	21.48	479.3497	456.3595	Testosterone undecanoate	C30 H48 O3
T5	(+)	21.65	343.2245	320.2353	(+/−)14,15-EpETrE	C20 H32 O3
T5	(+)	21.66	411.2861	388.297	1*α*,25-Dihydroxy-23,24-dinorvitamin D3	C25 H40 O3
T5	(+)	21.94	125.0381	248.0616	Dapsone	C12 H12 N2 O2 S
T6	(−)	17.77	248.1973	230.1635	Leu Val	C11 H22 N2 O3
T6	(−)	17.78	243.1331	242.1258	Hydroxyamobarbital	C11 H18 N2 O4
T6	(−)	21.56	839.4909	821.4566	Tacrolimus metabolite M-VIII	C43 H67 N O14
T6	(−)	21.65	498.9004	497.8933	Perflubron	C8 Br F17
T6	(+)	21.66	498.9008	497.8937	Perflubron	C8 Br F17
T6	(+)	21.95	125.0378	248.0611	Dapsone	C12 H12 N2 O2 S
T7	(−)	1.2	179.9883	141.0251	5-Acetyl-4-methylthiazole	C6 H7 N O S
T7	(−)	17.77	248.1964	230.1627	Leu Val	C11 H22 N2 O3
T7	(−)	21.65	498.9009	497.8938	Perflubron	C8 Br F17
T7	(+)	17.51	301.1413	278.152	Phthalic acid mono-2-ethylhexyl Ester	C16 H22 O4
T7	(+)	17.77	192.1362	169.1473	Lupinine	C10 H19 N O
T7	(+)	17.77	248.2013	247.194	Lycopodine	C16 H25 N O
T7	(+)	20.14	282.2793	281.2721	Oleoyl amine	C18 H35 N O
T7	(+)	21.44	411.2876	388.298	1*α*-hydroxy-21-nor-20-oxavitamin D3	C25 H40 O3
T7	(+)	21.45	343.2242	320.235	(+/−)14,15-EpETrE	C20 H32 O3
T7	(+)	21.62	343.224	320.2347	(+/−)14,15-EpETrE	C20 H32 O3
T7	(+)	21.64	411.2862	388.297	1*α*,25-Dihydroxy-23,24-dinorvitamin D3	C25 H40 O3
T7	(+)	22.15	167.011	128.0479	6-Hydroxy-2-hexynoic acid	C6 H8 O3
T7	(+)	22.74	179.9885	141.0251	5-Acetyl-4-methylthiazole	C6 H7 N O S
T8	(−)	16.01	243.1334	242.1261	Hydroxyamobarbital	C11 H18 N2 O4
T8	(−)	17.77	243.1333	242.126	Hydroxyamobarbital	C11 H18 N2 O4
T8	(+)	17.76	248.1967	230.1631	Leu Val	C11 H22 N2 O3
T8	(+)	17.77	192.1347	174.1013	Val Gly	C7 H14 N2 O3
T8	(+)	21.61	395.2916	356.3286	(+)-3-hydroxy behenic	C22 H44 O3
T8	(+)	21.67	909.5463	886.5559	Glc-GP(18:0/20:4(5Z,8Z,11Z,14Z))	C47 H83 O13 P
T8	(+)	21.93	125.0388	248.0632	Dapsone	C12 H12 N2 O2 S
T9	(−)	0.92	104.1065	103.0992	2-Amino-3-methyl-1-butanol	C5 H13 N O
T9	(−)	0.97	176.0109	137.0479	p-aminobenzoic acid	C7 H7 N O2
T9	(−)	0.98	293.0644	254.1012	Dyphylline	C10 H14 N4 O4
T9	(−)	0.99	160.0375	137.0483	3-Pyridylacetic acid	C7 H7 N O2
T9	(−)	1.02	203.0545	180.0654	Theobromine	C7 H8 N4 O2
T9	(−)	1.03	435.0635	396.0998	4-Hydroxylevamisole glucuronide	C17 H20 N2 O7 S
T9	(−)	1.05	265.0252	226.0621	Anthralin	C14 H10 O3
T9	(−)	1.13	230.0935	191.1302	Diethyltoluamide	C12 H17 N O
T9	(−)	1.19	287.1111	264.122	1-(5-Ketohexyl)-3-methylxanthine	C12 H16 N4 O3
T9	(−)	1.21	268.1553	267.1479	Metoprolol acid	C14 H21 N O4
T9	(−)	1.26	282.1712	281.1639	Benzenepropanoic acid	C15 H23 N O4
T9	(−)	1.98	362.2078	361.2005	Phe Val Pro	C19 H27 N3 O4
T9	(−)	2.03	362.242	344.2085	Oxyphencyclimine	C20 H28 N2 O3
T9	(−)	2.28	411.2	388.2101	Methyl 10,12,13,15-bisepidioxy-	C19 H32 O8
					16-Hydroperoxy-8E-octadecenoate	
T9	(−)	2.28	409.185	391.1507	Asn Met Gln	C14 H25 N5 O6 S
T9	(−)	2.45	621.217	620.2091	Diethylstilbestrol diglucuronide	C30 H36 O14
T9	(−)	2.89	480.225	479.2173	Gln Phe Trp	C25 H29 N5 O5
T9	(−)	3.23	480.225	479.2173	Gln Phe Trp	C25 H29 N5 O5
T9	(−)	3.5	601.155	578.1652	Rhoifolin	C27 H30 O14
T9	(−)	5.2	395.174	377.1404	Ofloxacin-N-oxide	C18 H20 F N3 O5
T9	(−)	5.26	395.206	377.1723	Gln Lys Cys	C14 H27 N5 O5 S
T9	(−)	5.69	205.062	182.0728	9-Hydroxyfluorene	C13 H10 O
T9	(−)	12.06	274.275	273.2672	C16 sphinganine	C16 H35 N O2
T9	(−)	12.19	318.3	317.2924	Phytosphingosine	C18 H39 N O3
T9	(−)	12.49	409.13	408.1232	Ichthynone	C23 H20 O7
T9	(−)	16.17	599.411	598.4032	Mytiloxanthin	C40 H54 O4
T9	(−)	20.08	797.52	774.5304	1,2 di-(9Z,12Z,15Z-octadecatrienoyl)-3-	C45 H74 O10
					O-beta-D-galactosyl-sn-glycerol	
T9	(−)	21.5	585.433	584.425	Myxol	C40 H56 O3
T9	(−)	21.5	601.427	600.4195	Capsanthin 3,6-epoxide	C40 H56 O4
T9	(−)	21.69	646.254	645.2443	Acarbose (Glucobay)	C25 H43 N O18
T9	(−)	21.49	607.29	568.3266	(3a,5b,7a)-23-carboxy-7-hydroxy-24-norcholan-3-yl,	C30 H48 O10
					b-D-glucopyranosiduronic acid	
T10	(−)	0.97	293.065	254.1022	Dyphylline	C10 H14 N4 O4
T10	(−)	1.03	265.025	226.0623	Anthralin	C14 H10 O3
T10	(−)	1.17	287.112	264.1228	1-(5-Ketohexyl)-3-methylxanthine	C12 H16 N4 O3
T10	(−)	2	362.243	344.2091	Oxyphencyclimine	C20 H28 N2 O3
T10	(−)	2.29	411.2	410.1925	Flumethasone	C22 H28 F2 O5
T10	(−)	2.29	409.185	408.1781	Trp Phe Gly	C22 H24 N4 O4
T10	(−)	2.45	621.218	620.2108	Diethylstilbestrol diglucuronide	C30 H36 O14
T10	(−)	2.92	480.225	479.2179	Gln Phe Trp	C25 H29 N5 O5
T10	(−)	3.25	480.225	479.2187	Gln Phe Trp	C25 H29 N5 O5
T10	(−)	3.64	264.159	246.1251	Santonin	C15 H18 O3
T10	(−)	5.23	395.207	377.1738	Gln Cys Lys	C14 H27 N5 O5 S
T10	(−)	12.19	318.302	317.2943	Phytosphingosine	C18 H39 N O3
T10	(−)	12.72	288.291	287.2833	C17 sphinganine	C17 H37 N O2
T10	(−)	13.14	288.29	287.2824	C17 sphinganine	C17 H37 N O2
T10	(−)	14.93	316.321	298.2873	11-Methyl-octadecanoic acid	C19 H38 O2
T10	(−)	14.97	272.295	254.2608	14-Methyl-8-hexadecen-1-ol	C17 H34 O
T10	(−)	15.51	304.286	286.2515	17-Hydroxy-heptadecanoic acid	C17 H34 O3
T10	(−)	16.17	599.409	598.4011	Mytiloxanthin	C40 H54 O4
T10	(−)	17.76	248.197	230.1627	Leu Val	C11 H22 N2 O3
T10	(−)	21.31	597.238	574.2481	p-hydroxy atorvastatin	C33 H35 F N2 O6
T10	(−)	21.48	585.433	584.4252	Myxol	C40 H56 O3
T10	(−)	21.63	607.287	568.3253	(3a,5b,7a)-23-carboxy-7-hydroxy-24-norcholan-3-yl,	C30 H48 O10
					b-D-Glucopyranosiduronic acid	
T10	(−)	21.69	469.368	468.36	1*α*,25-Dihydroxy-26,27-dimethyl-22,22,23,23-	C31 H48 O3
					tetradehydro-24a,24b-dihomovitamin D3	
T10	(−)	21.76	469.368	468.357	1*α*,25-Dihydroxy-26,27-dimethyl-22,22,23,23	C31 H48 O3
					-tetradehydro-24a,24b-dihomovitamin D3	
T10	(−)	21.77	429.375	428.367	25-hydroxy-26,27-dimethylvitamin D3/	C29 H48 O2
					25-hydroxy-26,27-dimethylcholecalciferol	
T10	(−)	22.48	607.287	606.277	Trandolapril glucuronide	C30 H42 N2 O11
T10	(+)	1.04	265.027	226.064	Anthralin	C14 H10 O3
T10	(+)	1.07	300.148	299.141	Metoclopramide	C14 H22 Cl N3 O2
T10	(+)	1.17	300.147	299.14	Metoclopramide	C14 H22 Cl N3 O2
T10	(+)	1.98	362.245	344.211	Oxyphencyclimine	C20 H28 N2 O3
T10	(+)	2.27	409.187	408.18	Trp Phe Gly	C22 H24 N4 O4
T10	(+)	5.22	395.208	377.175	Gln Cys Lys	C14 H27 N5 O5 S
T10	(+)	11.98	274.254	273.246	10Z,13Z,16Z-nonadecatrienenitrile	C19 H31 N
T10	(+)	12.15	230.248	212.214	Myristaldehyde	C14 H28 O
T10	(+)	12.17	318.3	317.293	Phytosphingosine	C18 H39 N O3
T10	(+)	17.76	243.139	242.131	6E,8E,14E-Hexadecatriene-10,12-diynoic acid	C16 H18 O2
T10	(+)	17.87	243.138	242.131	6E,8E,14E-Hexadecatriene-10,12-diynoic acid	C16 H18 O2
T10	(+)	18.22	419.281	418.273	Simvastatin	C25 H38 O5
T10	(+)	21.29	645.275	606.312	1,25-Dihydroxy-20S-21-(3-hydroxy-3-methylbutyl)	C32 H44 F6 O4
					-23-yne-26,27-hexafluorovitamin D3	
T10	(+)	21.7	646.258	645.249	Acarbose (Glucobay)	C25 H43 N O18
